# ﻿*Achnanthidiumbratanense* sp. nov. (Bacillariophyceae, Achnanthidiaceae), a new diatom from the Lake Bratan (Bali, Indonesia)

**DOI:** 10.3897/phytokeys.188.77882

**Published:** 2022-01-21

**Authors:** Dmitry A. Kapustin, Anton M. Glushchenko, Maxim S. Kulikovskiy

**Affiliations:** 1 Timiryazev Institute of Plant Physiology, Russian Academy of Sciences, 127276, Moscow, Russia Timiryazev Institute of Plant Physiology, Russian Academy of Sciences Moscow Russia

**Keywords:** Indonesia, monoraphid diatoms, morphology, new species

## Abstract

A new species, *Achnanthidiumbratanense*, is described from Lake Bratan, located on the island of Bali (Indonesia). The morphology of this species was analyzed with light (LM) and scanning electron microscopy (SEM). *A.bratanense* is characterized by linear-elliptic to nearly elliptic valves with convex margins and rounded, broadly subcapitate apices. The striae of this species are hardly discernable under LM; they are weakly radiate throughout the valve and composed of one to four large transapically elongated areolae of different length and shape. The most similar taxon to *A.bratanense* is *A.macrocephalum*, a species described from Sumatra, another Indonesian island. The differences of *A.bratanense* from similar taxa are discussed.

## ﻿Introduction

The genus *Achnanthidium* Kützing, 1844 is one of the largest genus among monoraphid diatoms. Although it had been described as a separate genus it was considered as a subgenus of *Achnanthes* Bory, from 1822 until the 90s (Round et al. 1990; Round and Bukhtiyarova 1996). Currently, *Achnanthidium* includes, according to different estimates, between 139 (Guiry and Guiry 2017) and nearly 200 species (Kociolek et al. 2021a). Revision of the genus continues up to now. Recently, two genera, namely *Gogorevia* (Kulikovskiy et al. 2020b) and *Gomphothidium* (Kociolek et al. 2021b) have been segregated from *Achnanthidium*.

The *Achnanthidium* taxa are common in different climatic zones all over the world (*e.g.*, Ponader and Potapova 2007; Wojtal et al. 2011; Novais et al. 2015; Karthick et al. 2017; Marquardt et al. 2017; Krahn et al. 2018; Yu et al. 2019). However, their identification is challenging because of the small size of these diatoms, often requiring examination using electron microscopy, and significant variability of diagnostic features (Ponader and Potapova 2007; Hlúbiková et al. 2011).

The number of publications dealing with freshwater diatoms from Indonesia is still rather low. The most comprehensive treatment was made by Hustedt (1937, 1942). Some of his new taxa have been re-examined (e.g., Hamsher et al. 2014; Kapustin et al. 2017, 2020; Kapustin and Kulikovskiy 2018; Wetzel et al. 2019; Kulikovskiy et al. 2020a). Also, a lot of new diatom species were described from Indonesian freshwaters over the last two decades (Bramburger et al. 2006; Kociolek et al. 2018; Kapustin et al. 2019, 2021; Kulikovskiy et al. 2019; Rybak et al. 2019), including two new *Achnanthidium* species (Tseplik et al. 2021a,b). The aim of this paper is to describe a new monoraphid species, *Achnanthidiumbratanense* sp. nov., from Lake Bratan located on the island of Bali, Indonesia.

## ﻿Materials and methods

A benthic sample containing *Achnanthidium* was collected from a volcanic Lake Bratan on 14 November 2010 (08°16.579'S, 115°09.985'E). For general characteristics of this lake see Green et al. (1978). Environmental variables were measured with a Hanna multiparameter probe meter (HANNA HI98128).

The sample was boiled in concentrated hydrogen peroxide (~37%) to dissolve the organic matter. It was then washed with deionized water four times at 12 h intervals. After decanting and filling with deionized water up to 100 ml, the suspension was spread on to coverslips and left to dry at room temperature. Permanent diatom slides were mounted in Naphrax. Light microscopic (LM) observations were performed with a Zeiss Scope A1 microscope equipped with an oil immersion objective (100×/n.a.1.4, differential interference contrast [DIC]) and Zeiss Axio-Cam ERc 5s camera. Valve ultrastructure was examined with a JSM-6510LV scanning electron microscope (Papanin Institute for Biology of Inland Waters RAS, Borok, Russia), operated at 10 kV and 11 mm distance. For scanning electron microscopy (SEM), parts of the suspensions were fixed on aluminum stubs after air-drying. The stubs were sputter coated with 50 nm of gold.

The original sample preserved with Lugol’s solution, as well as cleaned material preserved with 96% ethanol, are housed at the Laboratory of Molecular Systematics of Aquatic Plants, K.A. Timiryazev Institute of Plant Physiology, Russian Academy of Sciences (Moscow, Russia).

## ﻿Results

### ﻿ Achnanthidium bratanense

Taxon classificationPlantaeCocconeidalesAchnanthidiaceae

Kapustin, Glushchenko & Kulikovskiy sp. nov.

FCB79B0C-E7D6-52BD-BB7A-653273C1909B

Figures 1 , 2

#### Description.

**Figure 1.**
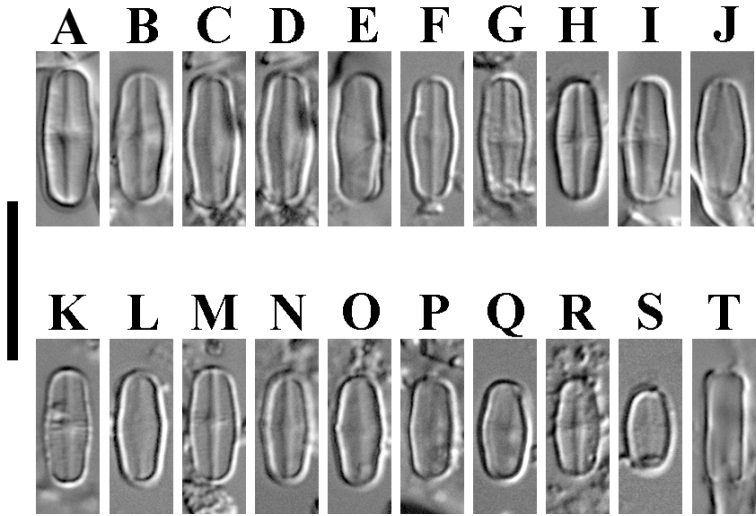
**A–T***Achnanthidiumbratanense* sp. nov. (LM). **A–S** size diminution series showing variation in valve outline **A** holotype specimen **A-D, F-I, K, M, N, R** raphe valves **E, J, L, O-Q, S** rapheless valves **T** frustule in girdle view. Scale bar: 10 μm.

***LM*** (Fig. 1A-T). Valves linear-elliptic to nearly elliptic with convex margins and rounded, broadly subcapitate apices. Frustules rectangular in girdle view and not bent (Fig. 1T). Length 5.0–8.7 µm, breadth 2.7–3.2 µm (n=32). In raphe valves axial area narrow, linear, slightly widening at center. Central area very small in raphe valves, outlined by shortened striae; central area in rapheless valves rhomboid (Fig. 1J). Raphe straight, filiform. In rapheless valves axial area expanded widening towards rhombic central area (Fig. 1E). Striae hardly discernable in LM, weakly radiate (Fig. 1A, H, K, M). Areolae indistinct in LM.

**Figure 2.**
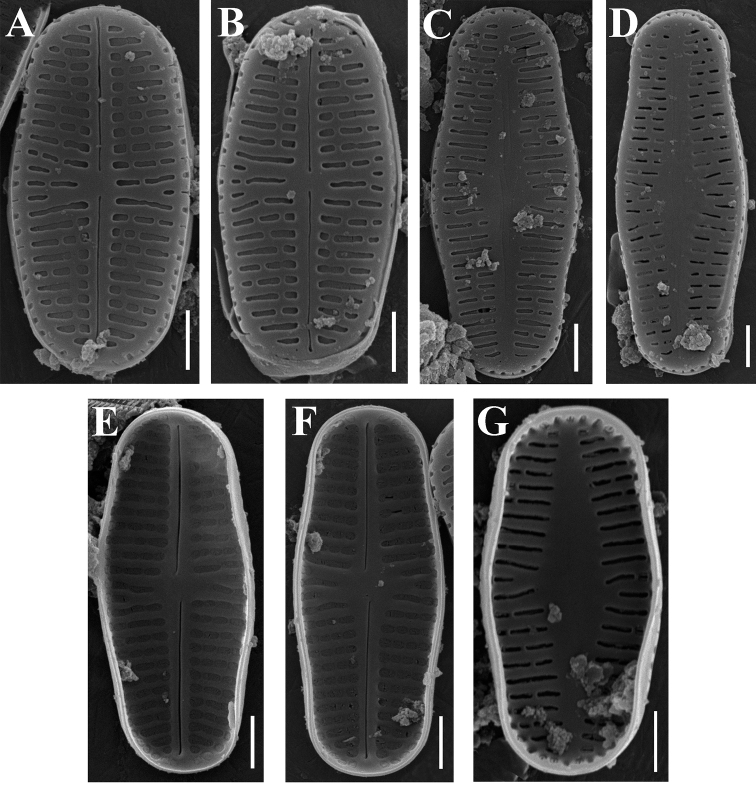
**A–G***Achnanthidiumbratanense* sp. nov. (SEM). **A, B** raphe valve, external view **C, D** rapheless valve, external view **E, F** raphe valve, internal view **G** rapheless valve, internal view. Scale bar: 1 μm.

***SEM*** (Fig. 2). Externally, raphe straight, filiform with drop-shaped proximal and distal raphe endings (Fig. 2A, B). Internally, proximal raphe endings deflected in opposite directions, distal raphe endings terminating in helictoglossae (Fig. 2E, F). Striae weakly radiate throughout the valve, 41–44 in 10 µm, and composed of one to four large transapically elongated areolae of different length and shape (from slit-like to irregularly rectangular). Areolae absent along valve margins; mantle with a single row of slit-like to almost rectangular areolae. Internally, areolae occluded by a hymen (Fig. 2E, F).

***Holotype*** (here designated): permanent slide No. MHA 01125, deposited at the Main Botanical Garden, Russian Academy of Sciences (MHA). Fig. 1A illustrates the holotype.

***Isotype*** (here designated): permanent slide No. 01125a, deposited in collection of Maxim Kulikovskiy, Timiryazev Institute of Plant Physiology, Russian Academy of Sciences.

#### Type locality.

Indonesia, Island of Bali, Lake Bratan, 08°16.579'S, 115°09.985'E, *leg.* I.I. Ivanov on 14 November 2010.

#### Etymology.

The specific epithet refers to the type locality, Lake Bratan.

#### Ecology.

*Achnanthidiumbratanense* together with *Gogoreviarinatii* were the most abundant species in the sample. Rarely single frustules of *Planothidium* sp., *Stauroneis* sp., *Cymbella* sp. and other diatoms were encountered. During sampling the temperature was recorded as 25.7 °C, pH as 7.82, and conductivity as 22 μS∙cm^−1^.

#### Distribution.

So far, this species is known from its type locality only.

## ﻿Discussion

Our new species is closely related to *Achnanthidiummacrocephalum* (Hustedt) Round & Bukhtiyarova, 1996. This taxon was described by Hustedt (1937) as Achnanthesminutissimavar.macrocephala from Sumatra. Recently, Wetzel et al. (2019) have re-examined Hustedt’s type material using both LM and SEM. Although the length and breadth of both species overlapped, *A.macrocephalum* is generally larger than *A.bratanense* (Table 1). However, the larger valves of *A.macrocephalum* have distinctly capitate apices. Despite the high abundance of *A.bratanense* we could not found such valves with capitate apices. Additionally, both taxa differ in striae density: *A.macrocephalum* has ca. 38 striae in 10 µm (Wetzel et al. 2019), whereas *A.bratanense* has 41–44 striae in 10 µm (see Table 1). In contrast to *A.macrocephalum*, the striae of *A.bratanense* is composed of 1–4 transapically elongated areolae. In *A.macrocephalum* the striae composed of 1 (smaller valves) or two (rarely 3) areolae (Wetzel et al. 2019). Also *A.bratanense* has weakly radiate striae throughout the valve whereas in *A.macrocephalum* the striae become parallel towards the valve ends.

**Table 1.** Comparison of morphological characteristics of *Achnanthidiumbratanense* sp. nov. and closely related taxa.

	*A.bratanense*	*A.macrocephalum*	A.rosenstockiivar.rosenstockii	A.rosenstockiivar.inareolatum	*Kolbesiasichuanenis*
Valve length, µm	5.0–8.7	7–12	6–14	9.6–15.1	10.8–14.1
Valve width, µm	2.7–3.2	2.5–3.2	3–4	4.2–5.1	3.2–3.7
Valve outline	linear-elliptic	linear-elliptic with convex margins	linear-lanceolate	linear-lanceolate	linear-lanceolate
Valve apices	subcapitate	rounded, broadly capitate	subcapitate	subcapitate	broadly capitate
Striae density	41–44	38	27–32	20	22–26
Number of areolae per stria	1–4	1–2(3)	2–4	1	1
Reference	This study	Wetzel et al. 2019	Krammer and Lange-Bertalot 2004	Krammer and Lange-Bertalot 2004; Yu et al. 2019	Yu et al. 2019

It should be noted that Hustedt (1942) reported Achnanthesminutissimavar.macrocephala from Lake Bratan on Bali and suggested that it might be widely distributed in the Indo-Malayan region. Unfortunately, he gave neither description nor images to support the written statement. It is very likely he actually observed *A.bratanense* instead of *A.macrocephalum*. Wetzel et al. (2019) supposed that Hustedt (1937) included in his description of *A.macrocephalum* two morphotypes.

Also *A.bratanense* is similar to several other taxa including A.rosenstockii (Lange-Bertalot) Lange-Bertalot var.rosenstockii, 2004, A.rosenstockiivar.inareolatum Lange-Bertalot, 2004, and *Kolbesiasichuanenis* P. Yu, Q-M. You & Q-X Wang, 2019 (Table 1). A.rosenstockiivar.rosenstockii is slightly wider than *A.bratanense* and the stria density is lesser (27–32 in 10 µm *vs.* 41–44 in 10 µm). A.rosenstockiivar.inareolatum differs from the type variety in having striae composed of a single macroareola. Probably, this taxon will be better to place in the genus *Karayevia* Round & Bukhtiyarova emend. Bukhtiyarova, 2006. From both A.rosenstockiivar.inareolatum and *Kolbesiasichuanenis*, *A.bratanense* differs in stria structure (number of areolae per stria) and stria density. Also, these taxa are significantly larger than *A.bratanense* (see Table 1).

Traditionally, three morphological groups are recognized within *Achnanthidium* (e.g. Novais et al. 2015; Karthick et al. 2017; Krahn et al. 2018; Yu et al. 2019): 1) *A.minutissimum* complex which is characterized by having straight external distal raphe ends, and striae density that increase towards the apex; 2) *A.pyrenaicum* complex which is characterized by having external distal raphe ends that deflect or hook to one side of the valve, and 3) *A.exiguum* complex have external distal raphe ends curved in opposite directions. Recently, the latter complex has been segregated into a new genus, *Gogorevia* (Kulikovskiy et al. 2020a). Although, *A.bratanense*, *A.macrocephalum* and *A.rosenstockii* can be placed in *A.minutissimum* complex based on the raphe structure, they have completely different striae structure and represent a separate morphological group. Interestingly, Pinseel et al. (2017) revealed 12 distinct lineages within *A.minutissimum* complex and one of them was described as the new species, *A.digitatum*Pinseel et al., 2017. Recently, Tseplik et al. (2021b) described from the ancient Lake Matano (island of Sulawesi, Indonesia) the new species, *A.gladius*Tseplik et al., 2021b, which was closely related to the latter taxon. Thus, further detailed study of the pore apparatus ultrastructure as well as molecular studies of *A.bratanense* and allied taxa will help to better understand the taxonomic status and phylogenetic placement of this morphological group.

## Supplementary Material

XML Treatment for Achnanthidium bratanense

## References

[B1] Bramburger AJ Haffner GD Hamilton PB Hinz F Hehanussa PE (2006) An examination of species within the genus *Surirella* from the Malili lakes, Sulawesi Island, Indonesia, with descriptions of 11 new taxa. Diatom Research 21(1): 1–56. 10.1080/0269249X.2006.9705650

[B2] Bukhtiyarova LN (2006) Additional data on the diatom genus *Karayevia* and a proposal to reject the genus *Kolbesia*. Nova Hedwigia. Beiheft 130: 85–96.

[B3] Green J Corbet SA Watts E Lan OB (1978) Ecological studies on Indonesian lakes. The montane lakes of Bali. Journal of Zoology 186(1): 15–38. 10.1111/j.1469-7998.1978.tb03354.x

[B4] Guiry MD Guiry GM (2017) *AlgaeBase*. https://www.algaebase.org/search/genus/detail/?genus_id=43670 [Accessed 2022–01–03]

[B5] Hamsher SE Graeff CL Stepanek JG Kociolek JP (2014) Variation in valve and girdle band morphology in freshwater *Denticula* (Bacillariophyceae) species: Implications for the systematic position of the genus including the description of *Tetralunata* gen. nov. (Epithemiaceae, Rhopalodiales). Plant Ecology and Evolution 147: 346–365. 10.5091/plecevo.2014.990

[B6] Hlúbiková D Ector L Hoffmann L (2011) Examination of the type material of some diatom species related to *Achnanthidiumminutissimum* (Kütz.) Czarn. (Bacillariophyceae). Algological Studies 136/137: 19–43. 10.1127/1864-1318/2011/0136-0019

[B7] Hustedt F (1937) Systematische und ökologische Untersuchungen über die Diatomeen-Flora von Java, Bali und Sumatra nach dem Material der Deutschen Limnologischen Sunda-Expedition. Systematischer Teil I. Archiv für Hydrobiologie (Supplement 15): 131–177.

[B8] Hustedt F (1942) Süßwasser-Diatomeen des indomalayischen Archipels und der Hawaii – Inseln. Internationale Revue der Gesamten Hydrobiologie und Hydrographie 42(1–3): 1–252. 10.1002/iroh.19420420102

[B9] Kapustin DA Kulikovskiy MS (2018) Transfer of *Stenopterobia* and *Surirella* taxa (Bacillariophyceae) described from the insular Southeast Asia to the genus *Iconella*. Nova Hedwigia. Beiheft 147: 237–245. 10.1127/nova-suppl/2018/019

[B10] Kapustin DA Kulikovskiy MS Kociolek JP (2017) *Celebesia* gen. nov., a new cymbelloid diatom genus from the ancient Lake Matano (Sulawesi Island, Indonesia). Nova Hedwigia. Beiheft 146: 147–155. 10.1127/1438-9134/2017/147

[B11] Kapustin DA Kociolek JP Glushchenko AM Kulikovskiy MS (2019) Four new species of *Cymbella* (Bacillariophyta) from the ancient Malili Lakes (Sulawesi Island, Indonesia). Botanicheskii Zhurnal 104(5): 766–780. 10.1134/S0006813619050065

[B12] Kapustin DA Kociolek JP Glushchenko AM Kulikovskiy MS (2020) A rediscovery of *Cymbellamirabilis* Hustedt, a rare endemic diatom, and description of *Alveocymba* gen. nov. Diatom Research 35(3): 281–287. 10.1080/0269249X.2020.1772888

[B13] Kapustin DA Glushchenko AM Kociolek JP Kulikovskiy MS (2021) *Encyonopsisindonesica* sp. nov. (Bacillariophyceae, Cymbellales), a new diatom from the ancient lake Matano (Sulawesi, Indonesia). PhytoKeys 175: 1–11. 10.3897/phytokeys.175.61044 33786008 PMC7990855

[B14] Karthick B Taylor JC Hamilton PB (2017) Two new species of *Achnanthidium* Kützing (Bacillariophyceae) from Kolli Hills, Eastern Ghats, India. Fottea 17(1): 65–77. 10.5507/fot.2016.020

[B15] Kociolek JP Kapustin DA Kulikovskiy MS (2018) A new, large species of *Gomphonema* Ehrenberg from ancient Lake Matano, Indonesia. Diatom Research 33(2): 241–250. 10.1080/0269249X.2018.1513868

[B16] Kociolek JP Blanco S Coste M Ector L Liu Y Karthick B Kulikovskiy M Lundholm N Ludwig T Potapova M Rimet F Sabbe K Sala S Sar E Taylor J Van de Vijver B Wetzel CE Williams DM Witkowski A Witkowski J (2021a) DiatomBase. *Achnanthidium* F.T. Kützing, 1844. http://www.diatombase.org/aphia.php?p=taxdetails&id=163531 [Accessed on 2021–10–09]

[B17] Kociolek JP You Q Yu P Li Y Wang Y Lowe R Wang Q (2021b) Description of *Gomphothidium* gen. nov., with light and scanning electron microscopy: A new freshwater monoraphid diatom genus from Asia. Fottea 21(1): 1–7. 10.5507/fot.2020.011

[B18] Krahn KJ Wetzel CE Ector L Schwalb A (2018) *Achnanthidiumneotropicum* sp. nov., a new freshwater diatom from Lake Apastepeque in El Salvador (Central America). Phytotaxa 382(1): 89–101. 10.11646/phytotaxa.382.1.4

[B19] Krammer K Lange-Bertalot H (2004) Bacillariophyceae 4. Teil: Achnanthaceae. Kritische Ergänzungen zu *Achnanthes* s. l., *Navicula* s. str., *Gomphonema*. Gesamtliteraturverzeichnis Teil 1–4. In: Ettl H Gärtner G Gerloff J Heynig H Mollenhauer D (Eds) Süßwasserflora von Mitteleuropa Band 2/4.Heidelberg, Elsevier GmbH, Spektrum Akademischer Verlag, 1–468.

[B20] Kulikovskiy M Maltsev Y Andreeva S Glushchenko A Gusev E Podunay A Ludwig TV Tusset E Kociolek JP (2019) Description of a new diatom genus *Dorofeyukea* gen. nov. with remarks on phylogeny of the family Stauroneidaceae. Journal of Phycology 55(1): 173–185. 10.1111/jpy.12810 30379324

[B21] Kulikovskiy M Kapustin D Glushchenko A Sidelev S Maltsev Y Gusev E Kezlya E Shkurina N Kuznetsova I Kociolek P (2020a) Morphological and molecular investigation of *Gomphonemalongissimum* and related taxa from Malili lakes (Indonesia) with comments on diatom evolution in ancient lakes. European Journal of Phycology 55(2): 147–161. 10.1080/09670262.2019.1664771

[B22] Kulikovskiy M Maltsev Y Glushchenko A Kuznetsova I Kapustin D Gusev E Lange-Bertalot H Genkal S Kociolek JP (2020b) *Gogorevia*, a new monoraphid diatom genus for *Achnanthesexigua* and allied taxa (Achnanthidiaceae) described on the basis of an integrated molecular and morphological approach. Journal of Phycology 56(6): 1601–1613. 10.1111/jpy.13064 32871027

[B23] Marquardt GC Costa LF Bicudo DC Bicudo CEM Blanco S Wetzel CE Ector L (2017) Type analysis of *Achnanthidiumminutissimum* and *A.catenatum* and description of *A.tropicocatenatum* sp. nov. (Bacillariophyta), a common species in Brazilian reservoirs. Plant Ecology and Evolution 150(3): 313–330. 10.5091/plecevo.2017.1325

[B24] Novais MH Jüttner I Van de Vijver B Morais MM Hoffmann L Ector L (2015) Morphological variability within the *Achnanthidiumminutissimum* species complex (Bacillariophyta): Comparison between the type material of *Achnanthesminutissima* and related taxa, and new freshwater *Achnanthidium* species from Portugal. Phytotaxa 224(2): 101–139. 10.11646/phytotaxa.224.2.1

[B25] Pinseel E Vanormelingen P Hamilton PB Vyverman W Van de Vijver B Kopalova K (2017) Molecular and morphological characterization of the *Achnanthidiumminutissimum* complex (Bacillariophyta) in Petuniabukta (Spitsbergen, High Arctic) including the description of *A.digitatum* sp. nov. European Journal of Phycology 52(3): 264–280. 10.1080/09670262.2017.1283540

[B26] Ponader KC Potapova MG (2007) Diatoms from the genus *Achnanthidium* in flowing waters of the Appalachian Mountains (North America): Ecology, distribution and taxonomic notes. Limnologica 37(3): 227–241. 10.1016/j.limno.2007.01.004

[B27] Round FE Bukhtiyarova L (1996) Four new genera based on Achnanthes (Achnanthidium) together with a re-definition of *Achnanthidium*. Diatom Research 11(2): 345–361. 10.1080/0269249X.1996.9705389

[B28] Round FE Crawford RM Mann DG (1990) The diatoms: biology and morphology of the genera. Cambridge University Press, 1–747.

[B29] Rybak M Solak CN Noga T Glushchenko A Williams DM Kulikovskiy M (2019) *Nupelabrevistriata* sp. nov. – a new, terrestrial diatom species from Southeast Asia. Diatom Research 34(4): 251–258. 10.1080/0269249X.2019.1698467

[B30] Tseplik ND Maltsev YI Glushchenko AM Kuznetsova IV Genkal SI Kociolek JP Kulikovskiy MS (2021a) *Achnanthidiumtinea* sp. nov. – a new monoraphid diatom (Bacillariophyceae) species, described on the basis of molecular and morphological approaches. PhytoKeys 174: 147–163. 10.3897/phytokeys.174.60337 33776528 PMC7979678

[B31] Tseplik ND Maltsev YI Glushchenko AM Kuznetsova IV Genkal SI Gusev ES Kulikovskiy MS (2021b) *Achnanthidiumgladius* sp. nov. (Bacillariophyceae) – a new monoraphid diatom species from Indonesia. PhytoKeys 187: 129–140. 10.3897/phytokeys.187.73913 35068971 PMC8709835

[B32] Wetzel CE Jüttner I Gurung S Ector L (2019) Analysis of the type material of Achnanthesminutissimavar.macrocephala (Bacillariophyta) and description of two new small capitate *Achnanthidium* species from Europe and the Himalaya. Plant Ecology and Evolution 152(2): 340–350. 10.5091/plecevo.2019.1628

[B33] Wojtal AZ Ector L Van de Vyver B Morales EA Blanco S Piatek J Smieja A (2011) The *Achnanthidiumminutissimum* complex (Bacillariophyceae) in southern Poland. Algological Studies 136/137: 211–238. 10.1127/1864-1318/2011/0136-0211

[B34] Yu P You Q-M Pang W-T Cao Y Wang Q-X (2019) Five new Achnanthidiaceae species (Bacillariophyta) from Jiuzhai Valley, Sichuan Province, Southwestern China. Phytotaxa 405(3): 147–170. 10.11646/phytotaxa.405.3.5

